# Recent Research Progress in the Structure, Fabrication, and Application of MXene-Based Heterostructures

**DOI:** 10.3390/nano12111907

**Published:** 2022-06-02

**Authors:** Ruxue Yang, Xiyue Chen, Wei Ke, Xin Wu

**Affiliations:** School of Chemical Engineering and Technology, Sun Yat-sen University, Zhuhai 519082, China; yangrx7@mail2.sysu.edu.cn (R.Y.); chenxy795@mail2.sysu.edu.cn (X.C.)

**Keywords:** two-dimensional materials, MXene, heterostructures

## Abstract

Two-dimensional (2D) materials have received increasing attention in the scientific research community owing to their unique structure, which has endowed them with unparalleled properties and significant application potential. However, the expansion of the applications of an individual 2D material is often limited by some inherent drawbacks. Therefore, many researchers are now turning their attention to combine different 2D materials, making the so-called 2D heterostructures. Heterostructures can integrate the merits of each component and achieve a complementary performance far beyond a single part. MXene, as an emerging family of 2D nanomaterials, exhibits excellent electrochemical, electronic, optical, and mechanical properties. MXene-based heterostructures have already been demonstrated in applications such as supercapacitors, sensors, batteries, and photocatalysts. Nowadays, increasing research attention is attracted onto MXene-based heterostructures, while there is less effort spent to summarize the current research status. In this paper, the recent research progress of MXene-based heterostructures is reviewed, focusing on the structure, common preparation methods, and applications in supercapacitors, sensors, batteries, and photocatalysts. The main challenges and future prospects of MXene-based heterostructures are also discussed to provide valuable information for the researchers involved in the field.

## 1. Introduction

Since the discovery of graphene, a growing family of two-dimensional materials has been widely studied by researchers due to their extraordinary properties in optical, electrical, thermal, and mechanical aspects, as well as the wide application potential [1,2,3,4,5,6,7,8,9,10]. 2D layered materials mainly include the graphene-like family [11,12,13], 2D transition metal chalcogenides [14,15,16], the 2D oxide family [17,18], and layered materials with other structures [19,20]. In 2011, Gogotsi et al. [21] discovered a type of highly conductive 2D nitride and carbide, which were called MXenes. MXenes, as one of the major subfamilies of MAX phase materials, are stripped by etching and ultrasonic treatment to generate 2D transition metal carbides, carbonitrides, or nitride layers [22]. During the preparation of MXenes, the surface is often rich with functional groups (-O, -OH, and -F), which can bring different properties to MXenes. As a new family of 2D nanomaterials, MXene shows excellent electrochemical, electronic, optical, and mechanical properties [23,24] due to its graphene-like structure and mixed covalent/metallic/ionic character, hydrophilicity, and unique metal conductivity. Therefore, MXene materials have attracted wide attention in the world and have developed rapidly in the past decade [25,26,27]. In general, MXene is prepared by selectively etching a layer in the MAX phase using hydrofluoric acid (HF). In order to improve the quality of MXene, simplify the experimental steps, and reduce the toxicity of the reagents, various preparation methods, such as thermal reduction, UV-induced etching, and alkali treatment, have emerged [28,29,30,31]. Currently, the family of MXene materials continues to develop and an increasing expansion of the applications in energy storage batteries, sensors, catalysts, and other fields has been witnessed [32,33,34,35].

To date, although MXene-based materials have been demonstrated to be widely used in different areas, there are still some challenges [36]. For example, the application of MXenes in fabricating flexible energy storage devices is limited due to the difficulty in achieving a good balance between mechanical and electrochemical properties [37]. The serious stacking phenomenon of MXenes impedes the diffusion of carriers in the vertical direction, lowering the specific capacity of MXenes under a high current density [38]. The poor oxidation resistance of MXenes in the application of the water-based flexible battery seriously affects its conductivity and cycling stability [39]. To overcome these shortcomings, different 2D nanomaterial structures are suggested to be spliced or stacked on top of each other, and as a result, many novel physical properties have been discovered.

In 2013, Geim and Grigoreva proposed for the first time a multilayer heterostructure, namely, van der Waals (vdW) heterostructure, which is formulated by using only the vertical vdW force between different layers to connect each 2D material and allow them to coexist in a stable way [40]. The discovery of 2D materials has breathed new life into the construction of heterogeneous structures. Traditional heterostructures are constructed by either doping homogenous materials, such as PN junctions of semiconductor silicon, or by epitaxial growth on lattice-matched substrate materials [41]. In this way, the material is severely limited, and serious dislocations and defects are easily formed at the interface, thus affecting the quality of the heterostructures. However, the 2D layered material has no dangling bonds on its surface, and different 2D atomic layers can be stacked together in selected order by means of weak van der Waals forces to form artificial heterostructures with atomically flat interfaces. Compared with traditional semiconductor heterostructures, 2D vdW heterostructures are not limited by lattice matching and material types, and can theoretically be stacked in any form (different types, angles, sequences, layers, etc.) like stacking wood [42,43,44]. The “arbitrary combination” of the van der Waals heterostructure allows these individual materials to be combined together while still maintaining the ultra-thin thickness [45]. Therefore, the emergence of vdW heterostructures offers a new structural platform for exploring new electronic and optoelectronic devices.

**Figure 1:**
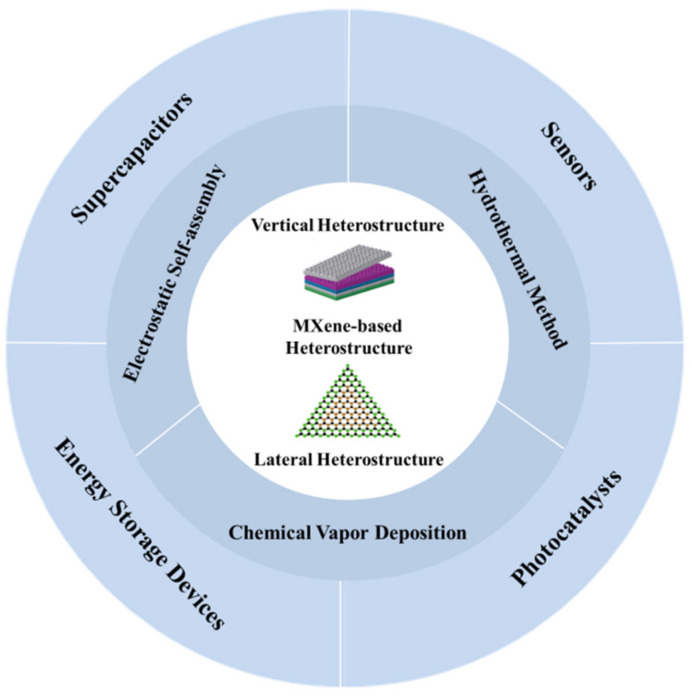
MXene-based heterostructures: the structure, fabrication, and application. The inserted figures are reprinted with permission from Ref. [40]. 2013, Springer Nature., and Ref. [52]. 2018, Springer Nature.

At present, numerous synthesis routes of MXene-based heterostructures have been proposed, and the synthesized heterostructures have been widely applied in the fields of supercapacitors, sensors, batteries, and photocatalysts [46,47,48]. Currently, some effort has been spent in summarizing the research progress of MXene materials, and a few articles have been written to review the research progress of MXene-based heterostructures in a certain application scenario, which can be complementary to this work [49,50,51]. In addition, the preparation and applications of MXene and MXene-based heterostructure materials are developing rapidly, and as a result, it is meaningful to write a timely review to summarize the current research status around this topic. This paper aims to give readers the latest research advances on MXene-based heterostructures. In this paper, the structure, preparation methods, and the applications of MXene-based heterostructures in supercapacitors, sensors, batteries, and photocatalysts are summarized (Figure 1). The main challenges and future prospects of MXene-based heterostructures are also analyzed.

## 2. Structure of MXene-Based Heterostructures

The family of MXene materials has a great variety and excellent electrochemical, optical, and mechanical properties. However, the realization of the applications of MXene materials is often limited by some inherent drawbacks. To overcome these issues, many novel heterostructures have been constructed based on the special optical and electrical properties of an individual 2D crystal, generating synergetic photoelectric properties, and therefore, wide attention has been received from researchers on this topic [53,54,55,56]. Generally, 2D heterostructures can be divided into two types: vertical heterostructures and lateral heterostructures. Two kinds of MXene-based heterostructures are described as following.

### 2.1. Vertical Heterostructures

**Figure 2:**
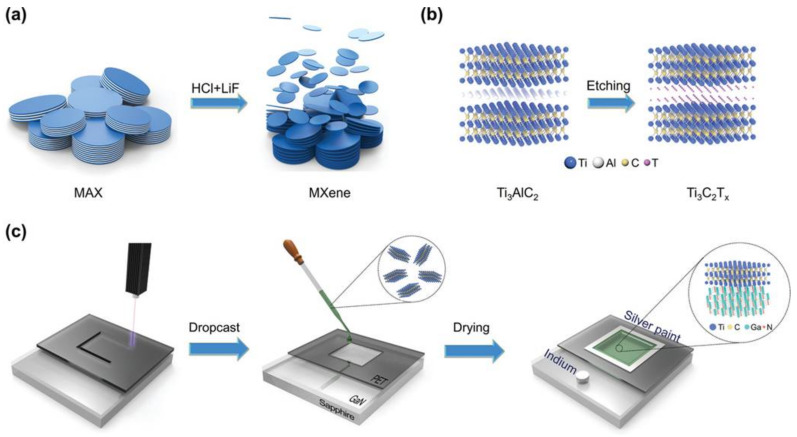
The synthesis process of Ti_3_C_2_T_x_ MXene and Ti_3_C_2_T_x_-GaN heterostructure. (**a**) Schematic of synthesis of MXene from MAX phase. (**b**) Schematic of the preparation of 2D Ti_3_C_2_T_X_ nanosheets. (**c**) Schematic of the fabrication of Ti_3_C_2_T_x_/GaN van der Waals heterostructure device. Reprinted with permission from Ref. [59]. 2021, John Wiley and Sons.

Vertical MXene-based heterostructures are synthesized by stacking independent monolayer 2D materials layer-by-layer through direct growth or the mechanical transfer method, which provides the heterostructure with a strong intralayer covalent bond and relatively weak interlayer vdW interaction, generating a system not limited by the lattice matching degree of the materials [57,58]. Due to the absence of suspended bonds and the weak vdW forces between the layered structures, the vertical MXene-based heterostructure can be easily constructed by stacking different materials. For instance, Yi et al. [59] fabricated MXene-GaN van der Waals heterostructures for photodetectors and LEDs. The synthesis process of the MXene-GaN van der Waals heterostructure is schematically illustrated in Figure 2. The devices based on MXene-GaN heterostructures exhibited good photodetection performance. Dai et al. [60] designed vertical 2D Ti_3_C_2_T_X_ MXene/V_2_O_5_ heterostructures by freeze-drying for the application of membrane electrodes. Vertical channels were formed in the heterostructures to promote rapid electron and ion transport throughout the electrode. Moreover, Yuan et al. [61] formed the BN/Ti_3_C_2_T_x_ van der Waals heterostructure for lithium-ion batteries by high-energy ball milling, which plays a series of roles in increasing the layer spacing, reducing the size of nanosheets, and maintaining the structural integrity. The experimental results showed that the heterostructure has excellent rate performance and long-term cycle stability.

Although vertical heterostructures have become one of the hottest research fields in recent years, there are two major problems limiting the applications of vertical heterostructures in various devices: (1) foreign pollutants are easily introduced during the preparation process; (2) the stacking direction is not controllable. The construction of lateral heterostructures can overcome these limitations.

### 2.2. Lateral Heterostructures

Lateral MXene-based heterostructures are generally prepared by seamlessly integrating 2D materials into one plane through direct growth, which can accurately control the direction and quality of the interface inside the 2D lateral heterostructures [62]. The 2D lateral heterostructure is connected by covalent bonds, which provide excellent intralaminar stability and improve the epitaxial quality.

**Figure 3:**
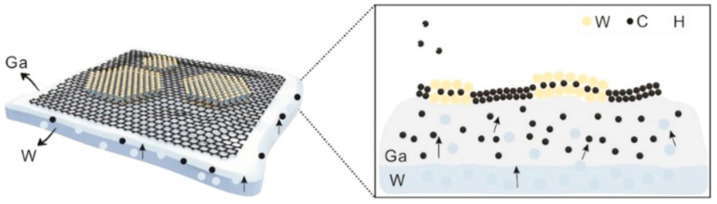
Schematic drawing and cross-view of 2D lateral WC-graphene (WC-G) heterostructures prepared by chemical vapor deposition (CVD). Reprinted with permission from Ref. [63]. 2017, Elsevier.

Compared with vertical MXene-based heterostructures, the construction of 2D lateral heterostructures ismore difficult in practice, and we cannot randomly choose the initial 2D materials to construct any heterostructure as we desire. Although the 2D lateral heterostructures are difficult to synthesize, the advantages of covalent bonding in the atomic plane and easy plane integration arouse people’s great interest. Zeng et al. [63] prepared 2D lateral WC-graphene (WC-G) heterostructures based on a versatile approach, which demonstrated excellent chemical stability and reactivity, as seen in Figure 3. Currently, there are limited studies on 2D lateral MXene heterostructures, but due to the special properties and significant application potential, more research efforts on this topic can be expected in the coming years.

## 3. Fabrication of MXene-Based Heterostructures

Two-dimensional heterostructures can be prepared by deterministic transfer methods, CVD epitaxial growth methods, and self-assembly [64]. The various synthesis approaches of 2D heterostructures directly affect their physical and chemical properties, thus affecting their application fields [65]. Generally, the deterministic transfer method and CVD epitaxial growth method are most often used to construct 2D heterostructures [66]. PDMS, PPC, and PMMA are commonly used in deterministic transfer methods. As for the CVD epitaxial growth method, it is suitable for both vertical heterostructures and lateral heterostructures [67]. By adjusting the temperature, composition, velocity, and direction of the flow, different types of heterostructures can be prepared. Currently, three major preparing methods have been proposed for constructing MXene-based heterostructures, namely, the hydrothermal method [68], electrostatic self-assembly method [69], and chemical vapor deposition [70].

### 3.1. Hydrothermal Method

The hydrothermal method [71] refers to the method of preparing materials by dissolving and recrystallizing powders with water as the solvent in a sealed pressure vessel. The hydrothermal method has the advantages of relatively mild operating conditions, high crystallinity of products, environmental friendliness, and good dispersity. In addition, the cost of hydrothermal synthesis is lower in terms of instrumentation, energy, and material precursors compared to gas and solid-phase methods. MXene is dispersed in the liquid phase with another material to obtain a heterostructure under hydrothermal conditions [72]. Under the conditions of high temperature and high pressure, this method is able to improve the activity and manipulate the functional groups at the surface of MXene.

In practical applications, MXene-based heterostructures with rich functions are usually required. A hydrothermal environment can control the functional groups on the surface of MXene-based heterostructures, so as to improve their activity. Qiao et al. [73] designed and fabricated Ti_3_C_2_/CdS heterostructures for use as highly efficient co-catalysts by a hydrothermal strategy. The characterization results showed that the Ti_3_C_2_/CdS heterostructure was spontaneously decorated with a large number of hydrophilic functional groups (-OH and -O). In addition, the CdS/Ti_3_C_2_ heterostructure with a cauliflower structure showed ultra-high visible light photocatalytic activity and has great application potential in the field of photocatalysis. Wang et al. [74] constructed a 1T-MoS_2_/Ti_3_C_2_ MXene heterostructure for a supercapacitor via the hydrothermal method and studied the electrochemical storage mechanism of the heterostructure. The experimental results showed that the supercapacitor-based 1T-MoS_2_/Ti_3_C_2_ MXene heterostructure has a high capacitance ratio and excellent rate performance, and maintains an excellent cycling stability after tens of thousands of cycles because of the synergistic effect between MoS_2_ and MXene.

**Figure 4:**
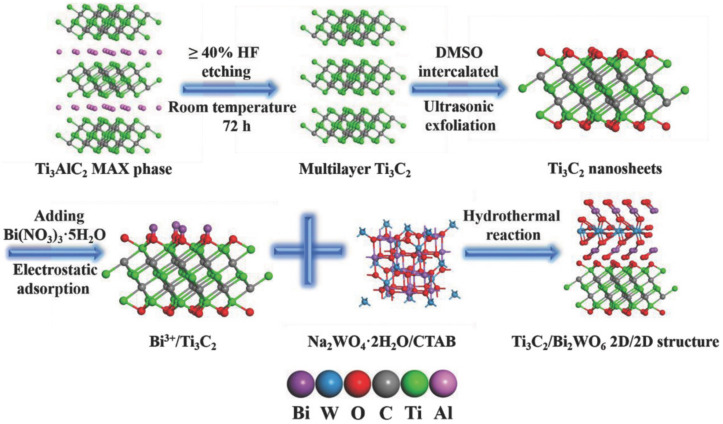
The synthetic process of the novel 2D/2D Ti_3_C_2_/Bi_2_WO_6_ heterostructure. Reprinted with permission from Ref. [75]. 2018, John Wiley and Sons.

Under the hydrothermal environment, the functional groups on the surface of MXenes are improved. Due to the electrostatic interaction and other effects, the second phase dispersed in the liquid phase can grow in situ on the surface of MXene, and the two kinds of materials are in close contact to form a heterostructure, which has strong interface interaction, excellent electron transfer ability, and can provide a large interface contact area at the interface. Cao et al. [75] successfully prepared a novel 2D/2D Ti_3_C_2_/Bi_2_WO_6_ heterostructure through a hydrothermal strategy. The synthesized Ti_3_C_2_/Bi_2_WO_6_ heterostructure showed an excellent ability forphotocatalytic reduction of CO_2_, which was mainly due to the improvement of the specific surface area and pore structure of the synthesized heterostructure, as well as the short charge-transfer distance and large interface contact area. Figure 4 indicates the synthetic process of the novel 2D/2D Ti_3_C_2_/Bi_2_WO_6_ heterostructure. Ye et al. [76] synthesized CdS/Ti_3_C_2_ heterostructures by the hydrothermal method to construct a Cu^2+^ sensor based on photoelectrochemical (PEC) detection. The results indicated that the sensor has a sensitive response, low detection limit, and great photocurrent signal, owing to the benefits from the improved carrier transport at the interface of the CdS/Ti_3_C_2_ heterostructure.

### 3.2. Electrostatic Self-Assembly Method

Electrostatic self-assembly [77] uses the electrostatic interaction of two nanomaterials with opposite charges in an aqueous solution for self-assembly, so as to form nanoscale ultra-thin polymer materials. Among many self-assembly methods, electrostatic self-assembly has a wide range of applications, owing to its simplicity and controllable thickness [78]. As a common method for constructing two-dimensional heterostructures, a variety of MXene-based heterostructures have been constructed via electrostatic self-assembly and have been applied in many fields [71]. However, electrostatic self-assembly is less stable due to the electrostatic interaction and hydrogen bonding.

**Figure 5:**
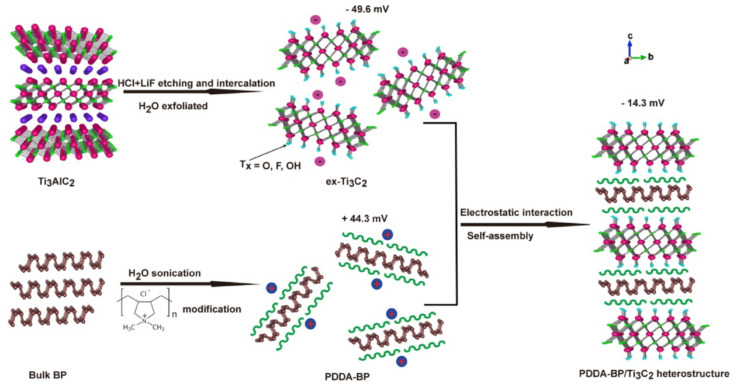
Schematic diagram of the synthesis process of PDDA−BP/Ti_3_C_2_ heterostructures. Reprinted with permission from Ref. [79]. 2019, Elsevier.

The layer-by-layer stacking of the layered structure can re-stack the nanosheets with different functional properties into heterogeneous structures, which undoubtedly makes full use of the characteristics of each heterogeneous component and presents superior electrochemical performance coordinated with the mechanical structure. Zhao et al. [79] designed and prepared molecular-level (PDDA-BP/Ti_3_C_2_) heterostructures for the sodium-ion battery through the electrostatic self-assembly process, as shown in Figure 5. It revealed that surface functional groups of -F, -O, and -OH in Ti_3_C_2_ play important roles to immobilize BP and that the PDDA-BP/Ti_3_C_2_ heterostructure provides effective chargetransfer and diffusion channels, thus exhibiting significantly improved electrochemical properties and structural stability. In 2019, Liu et al. [80] reported the heterostructure synthesis of MXenes@C for magnesium-ion storage via electrostatic interactions between negatively charged 2D MXene nanosheets and positively charged 3D carbon nanospheres, which could effectively prevent the re-stacking of MXene nanosheets, so as to promote the transmission of electrolytes and shorten the ion diffusion path. Tests revealed that the magnesium-ion storage battery exhibits high reversible specific capacity, outstanding rate capacity, and excellent cycle stability. Moreover, Wen et al. [81] prepared three-dimensional hierarchical nMOF-867/Ti_3_C_2_T_x_ heterostructures for lithium-sulfur batteries via electrostatic self-assembly. The lithium-sulfur battery based on the nMOF-867/Ti_3_C_2_T_x_ heterostructures had strong conductivity and could reduce the volume expansion during cycling. This work provided the inspiration for preparing high-performance lithium-sulfur batteries based on MXene-based heterostructures. Electrostatic self-assembly uses MXenes with functional groups on the surface and materials with opposite surface charges to construct heterostructures by electrostatic attraction. As a simple, easily operated method, it can effectively open the middle layer and prevent the re-stacking of MXene nanosheets, thus providing an effective charge-transfer channel and shortening the ion diffusion path.

In recent years, due to its simplicity, electrostatic self-assembly has also been adopted to synthesize photocatalysts with high photocatalytic activity. Hu et al. [82] synthesized 2D/2D Ti_3_C_2_/porous g-C_3_N_4_ (TC/PCN) photocatalysts through a facile electrostatic self-assembly method by integrating the merits of g-C_3_N_4_ and Ti_3_C_2_. The synthesized heterostructures exhibited exceptional performance compared with pure PCN and the observed activity had no significant decrease after four cyclic experiments. In another experiment, boron-doped graphite carbonitride (g-C_3_N_4_) and few-layer Ti_3_C_2_ MXene were combined to construct heterostructures by electrostatic self-assembly for enhanced photocatalytic reduction of CO_2_ [83]. The optimized composite structure had excellent photocatalytic activity and stability. The yields of CO and CH_4_ were 3.2 times and 8.9 times higher than that of a bare g-C_3_N_4_, respectively. Zhuang et al. [84] successfully prepared TiO_2_/Ti_3_C_2_ heterostructures by the electrostatic self-assembly technique. The maximum hydrogen production rate was 2.8 times larger than that of pure TiO_2_ nanofibers, and the nanocomposite maintained a good hydrogen production cycle capacity, owing to the heterogeneous interface between TiO_2_ and Ti_3_C_2_ nanosheets.

### 3.3. Chemical Vapor Deposition (CVD)

Chemical vapor deposition mainly uses one or several gas-phase compounds or elements containing film elements to generate film on the substrate surface by chemical reaction. In the CVD process, parameters such as pressure, temperature, gas flow rate, and catalyst type can be adjusted to achieve fine control of the size, layer number, morphology, and quality of 2D lattices. Chemical vapor deposition (CVD) has been widely used in the preparation of heterostructures owing to its low cost, extensibility, and full controllability. The synthesis of vertical and lateral heterostructures from different 2D materials can result in many excellent physical properties and has been applied in the fields of batteries, catalysts, and sensors, which largely depends on the arrangement, quality, and interface of the combined 2D layered crystals.

Based on a one-step CVD method, Zeng et al. [63] reported the embedding of a 2D WC crystal into graphene to fabricate 2D WC-graphene lateral heterostructures on metal gallium (Ga) by integrating a liquid metal-based co-segregation strategy. The as-synthesized heterostructure exhibited excellent catalytic potential, which provided a good reference for fabricating other high-quality in-plane 2D transition metal carbide-based structures. In general, the heterostructures of graphene and other 2D materials are fabricated by stacking, which leads to random arrangement, weak interface interactions, and inevitable interface pollutants during the preparation process. Xu et al. [85] constructed high-quality graphene/α-Mo_2_C crystal vertical heterostructures with uniformly well-aligned lattice orientation and strong interface coupling by a two-step CVD method. During the two-step CVD, the authors maintained a constant atmosphere to avoid defects, thus forming high-quality heterostructures.

**Figure 6:**
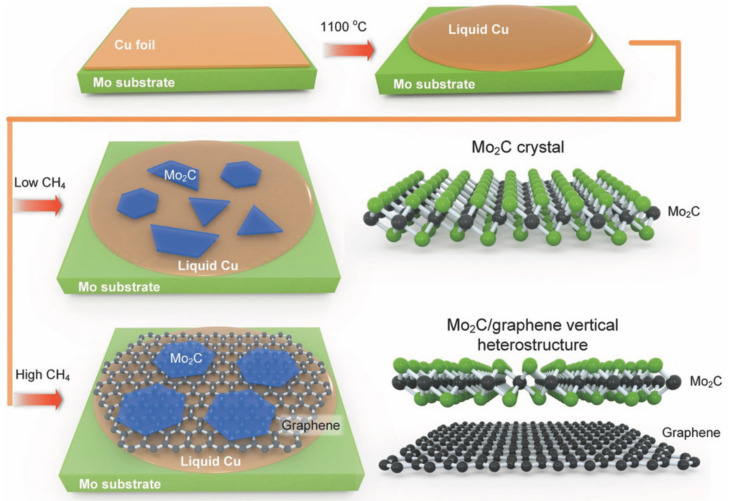
Schematic display of the growing process of Mo_2_C/graphene heterostructure at low and high CH_4_flow rates. At low CH_4_ concentration, Mo_2_C crystals with different shapes and thicknesses are randomly distributed on the copper surface, while at high CH_4_ concentration, hexagonal thin Mo_2_C sheets are mainly grown on graphene. Reprinted with permission from Ref. [86]. 2017, John Wiley and Sons.

Fabrication of vertically stacked 2D heterostructures usually requires a site-point transfer process. Geng et al. [86] directly synthesized large-area and uniform 2D Mo_2_C-graphene heterostructures on the in-situ grown graphene substrate in one step by ambient pressure CVD (Figure 6), which circumvents the need for transfer processes. Due to the effective interfacial charge-transfer kinetics, the Mo_2_C/graphene heterostructure showed higher catalytic activity than the Mo_2_C-only catalyst in a hydrogen evolution reaction (HER). In another study, Sun et al. [87]. explored the growth mechanism of 2D Mo_2_C on liquid metals. The characterization of AFM showed that Mo_2_C is grown on Au substrate by a sunk growth mode. In addition, the controllable synthesis of graphene/Mo_2_C heterostructure was mainly realized by adjusting the hydrocarbon ratio.

At present, chemical vapor deposition (CVD) has been widely used to prepare vertical and lateral heterostructures. Compared with the stacking method, the MXene-based heterostructures prepared by CVD can obtain a very clean interface. In addition, high-quality MXene-based heterostructures can be synthesized by carefully controlling the preparation parameters. What’s more, the as-synthesized heterostructures have a strong interface interaction.

Among the three common synthesis approaches of MXene-based heterostructures, the hydrothermal method has the advantages of relatively mild operating conditions, environmental friendliness, good dispersion, and low cost. At the same time, the activity of heterostructures can be improved and the functional groups on the surface of MXenes can be manipulated in the hydrothermal environment. Electrostatic self-assembly is widely used because of its simple preparing procedures and controllable thickness. However, the surface of the constituent materials needs to be pretreated. In the CVD process, the parameters such as pressure, temperature, gas flow rate, and catalyst type can be adjusted to realize the fine control of the size, layer number, morphology, and quality of MXene-based heterostructures, and CVD is applicable to synthesize both vertical and lateral heterostructures. The high-quality heterostructures synthesized by the aforementioned three methods are able to provide a large interface contact area and a short charge-transfer distance at the interface, as well as prevent the stacking of MXene layers, generating an improved interfacial carrier transport.

## 4. Applications of MXene-Based Heterostructures

The advances of novel 2D nanomaterials and related nanotechnology have continuously promoted the fast development of areas such as sustainable energy conversion [88], storage equipment [89], and flexible electronic devices [90]. Compared with other nanomaterials, MXenes have attracted increased attention due to their excellent electrical conductivity and hydrophilicity, superior electrochemical performance, large specific capacitance, adjustable layer structure, and controlled interface chemistry [91]. However, MXenes are severely limited by these problems: sluggish reaction kinetics, limited active sites, low material utilization efficiency, and severe stacking in practical applications. The MXene-based heterostructures can be constructed to optimize the performance of MXenes. At present, heterostructures based on MXenes have been widely used in the fields of supercapacitors, sensors, energy storage batteries, and photocatalysts.

### 4.1. Supercapacitors

The supercapacitor is recognized as a new type of energy storage device. Due to the features of a fast charge-discharge process and good energy storage capability, the supercapacitor presents the advantages of the traditional capacitor and rechargeable battery. MXene is suitable for electrode materials because of its unique metal conductivity, high conductivity, and surface hydrophilicity [92]. MXene materials can produce high energy and power density, as well as fast electrochemical charge storage, improved mechanical stability, and shortened ion diffusion paths between positive and negative poles [93]. Therefore, MXenes are promising candidates in the field of supercapacitors. However, MXene flakes tend to be stacked again during electrode fabrication, which is not conducive to the rapid diffusion of ions in the vertical direction and affects their specific capacity under high current density [94]. The heterostructures constructed by MXene and a variety of nanomaterials can effectively improve the capacitance electrochemical behavior.

**Figure 7:**
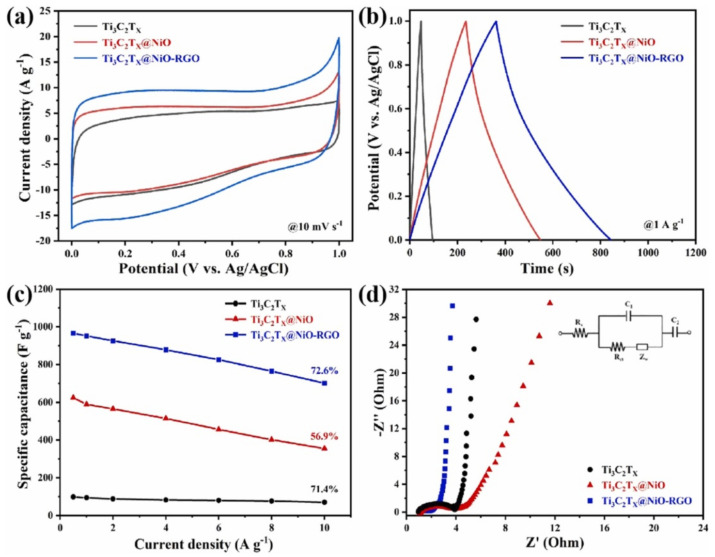
Application of MXene heterostructures in supercapacitors. (**a**) CV curves of Ti_3_C_2_T_x_, Ti_3_C_2_T_x_@NiO and Ti_3_C_2_T_x_@NiO−RGO hydrogel at the potential scan rate of 10 mV·s^−1^. (**b**) GCD curves of Ti_3_C_2_T_x_, Ti_3_C_2_T_x_@NiO and Ti_3_C_2_T_x_@NiO−RGO hydrogel at the current density of 1 A·g^−1^. (**c**) Specific capacitance vs. current density plot of Ti_3_C_2_T_x_, Ti_3_C_2_T_x_@NiO and Ti_3_C_2_T_x_@NiO−RGO hydrogel. (**d**) Nyquist impedance plots of Ti_3_C_2_T_x_, Ti_3_C_2_T_x_@NiO and Ti_3_C_2_T_x_@NiO−RGO hydrogel at 0 V, with the equivalent circuit shown in the insert. Reprinted with permission from Ref. [96]. 2022, Elsevier.

The low-rate performance caused by the sheet self-stacking of the traditional MXene electrode limits its electrochemical application to a certain extent. Luo et al. [95] reported the preparation of a reduced graphene oxide/Ti_3_C_2_T_x_ electrode for a supercapacitor. The fabricated composite electrode exhibited outstanding electrochemical performance and mechanical flexibility, and still maintained excellent cycle stability after 32,000 charge-discharge cycles. In a different study, a 3D hierarchical Ti_3_C_2_T_x_@NiO-RGO heterostructure was synthesized by chemical bath deposition and thermal annealing for application as SCs [96]. The Ti_3_C_2_T_x_@NiO-RGO electrode showed excellent electrochemical performance, with ultra-high specific capacitance, excellent cycle life, and high energy density. Due to the synergistic interaction between Ti_3_C_2_T_x_ and NiO, the heterostructure had rich ion diffusion and electron transfer pathways (as shown in Figure 7). Converting the planar structure of 2D MXene film into an interpenetrating network with open or porous structures is an effective way to construct high-performance supercapacitors. Xia et al. [97] prepared a MXene/SiC heterostructure by ionization-bombardment assisted deposition for a micro-supercapacitor. The electrochemical test results showed that the specific capacity of the MXene/SiC heterostructure was as high as 97.8 mF·cm^−2^ at the current density of 1 A·cm^−2^, which was mainly due to the unique three-dimensional network structure of the heterostructure. By modifying the MXene surface through a SiC nanostructure, the ion storage space is greatly increased. Wang et al. [98] reported the fabrication of a hydrophilic 2D/2D NiMoO_4_/MXene heterostructure for supercapacitors with an interconnected porous network. The 2D/2D NiMoO_4_/Ti_3_C_2_T_x_ heterostructure exhibited a high specific capacity of 545.5 C·g^−1^ at a current density of 0.5 A·g^−1^ and an excellent cycling life, which could maintain 72.6% of the initial capacitance at a current density of 5 A·g^−1^ after 10,000 cycles thanks to the excellent conductivity and hydrophilicity of Ti_3_C_2_T_x_ and the synergistic effect between NiMoO_4_ and Ti_3_C_2_T_x_. In addition, Wang et al. [74] prepared 1T-MoS_2_/Ti_3_C_2_ MXene heterostructures by magneto-hydrothermal synthesis and investigated the electrochemical storage mechanisms. The supercapacitor achieved excellent rate performance and expanded ion storage space as a result of the synergistic interaction between MoS_2_ and Ti_3_C_2_.

### 4.2. Batteries

In recent years, batteries have been seen as a hotly contested research area [99]. They have already been widely used in tiny electric cars and consumer electronics. As a result, in addition to long life cycles, there is a need for new and highly efficient electrodes with the required power, compactness, and energy density [100]. MXene can be used as an electrode material due to its high metal conductivity, rich surface functional groups, and controllable layer spacing. However, due to the strong van der Waals interaction and hydrogen bonding between adjacent nanosheets, the aggregation and self-stacking of multilayer MXene nanosheets usually occur during electrode fabrication, which largely limits the accessibility of electrolyte ions and prevents the full utilization of their surface functions. In order to overcome this shortcoming, the method of constructing MXene-based heterostructures has been proposed. Compared with other structural materials, MXene-based heterostructures can provide a stable volume buffer space and can take full advantage of the properties of two components to enhance the electrochemical performance [101].

**Figure 8:**
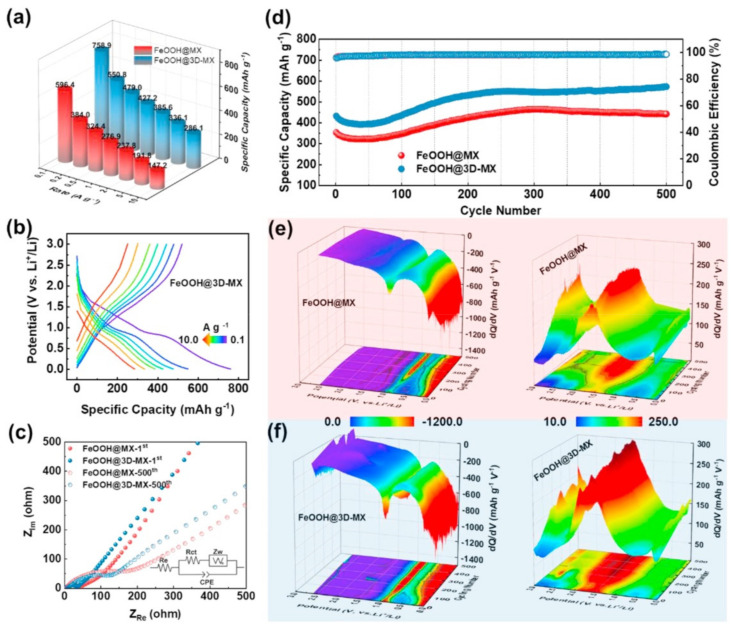
Electrochemical performance of FeOOH@MX and FeOOH@3D−MX electrodes for usage in Li^+^ storage battery: (**a**) rate properties, (**b**) the discharging and charging properties of FeOOH@3D−MX when current densities change from 0.1 to 10 A·g^−1^, (**c**) Nyquist plots (the equivalent circuit is shown in the inset), (**d**) cyclic performance at the condition of 500 mA·g^−1^, and (**e**,**f**) corresponding dQ/dV performance under long-period cycling conditions. Reprinted with permission from Ref. [103]. 2021, Elsevier.

Integrating different materials to build heterostructures can combine their advantages and achieve synergistic effects. In one work, Cao et al. [102] prepared a new self-assembled ternary heterostructure based on transition metal selenides, MXene nanosheets, and nitrogen-rich carbonaceous nanoribbons (CNRibs) with ultrafast ion transport properties for sodium and potassium-ion storage. This MXene-based ternary heterostructure could effectively prevent the re-stacking of 2D materials and increase the inherent conductivity. Most importantly, the heterostructure provided ultra-fast interface ion transport pathways and additional surface and interface storage sites, thereby improving the performance of high-interest storage in SIB and PIB applications. Recently, Ruan et al. [103] prepared FeOOH/MXene heterostructures for lithium-ion batteries by the one-step simple immersion method. The lithium-ion battery showed excellent high-rate performance and stable lithium-ion storage performance, as shown in Figure 8. It is because the FeOOH/MXene heterostructure could enhance the charge-transfer efficiency and provide a stable interface structure. Fabricating heterostructures is a growing strategy to overcome the problems of poor cyclic stability and electrode kinetic retardation caused by the violent oscillations of lithium polysulfides (LiPSs) during the commercialization of Li-S batteries, but the underlying design mechanism is still an unsolved problem. Gao et al. [104] used first-principles calculations to illustrate how the fabrication of heterostructures can effectively transform LiPSs and improve sulfur utilization based on the Sc_2_Co-MXene/h-BN heterostructure. The results showed that the heterostructures exhibited enhanced LiPS anchoring ability, had a diffusion barrier as low as 0.71 eV, and exhibited a decomposition barrier of 1.29 eV for the Li_2_S cluster, and therefore, the Li-S batteries effectively achieved high capacity and coulomb efficiency.

**Figure 9:**
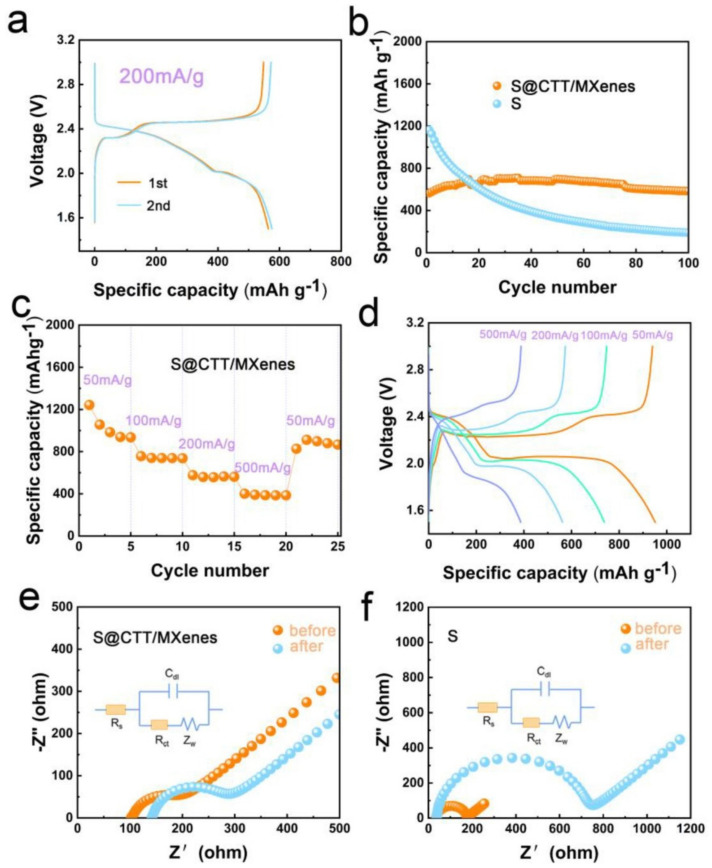
Electrochemical performance of S@CTT/MXene−based electrode. (**a**) CV properties at a condition of 200 mA·g^−1^. (**b**) Specific capacity under different cycling numbers and (**c**) evolution of rate capabilities of S@CTT/MXene. (**d**) The influence of current density, and (**e**,**f**) EIS performance of S@CTT/MXene−based electrode. Reprinted with permission from Ref. [106]. 2022, Elsevier.

In addition, in recent years, wearable electronic devices bring an important impact to people’s lives, in which flexible batteries play an irreplaceable role. MXene-based heterostructures can integrate the merits of different materials to achieve enhanced mechanical properties with isotropic characteristics, which is able to ensure the structure stability, as well as provide a new idea and paradigm for the construction of flexible electrodes. Zhang et al. [105] constructed the MoS_2_/MXene heterostructure based on MoS_2_ nanoflowers, MXene micro-nano materials, and hollow carbonized kapok fibers (CKFs) for a high-speed sodium-ion battery by the hydrothermal method. The MoS_2_/MXene heterostructure made full use of the advantages of MoS_2_ and MXene, showing excellent coulomb efficiency and high capacitance thanks to the layered, hollow, and porous heterostructure. In order to obtain the high energy density and high safety of Li-S batteries, Feng et al. [106] prepared all-solid-state lithium-sulfur batteries based on COF-derived N-doped porous carbon and two-dimensional MXene. Taking advantage of the synergistic effect of porous and electronic conductive CTT, flexible MXene, and solid-state electrolytes, the all-solid-state lithium-sulfur battery showed outstanding energy density, high safety, and excellent cycle stability. Figure 9 shows the electrochemical performance of the S@CTT/MXene anode. Zhao et al. [107] prepared 2D MXene/graphene heterostructures for flexible and conductive paper electrodes, which were directly used as anodes for sodium-ion storage without adhesives, conductive additives, or collectors, showing excellent cycle stability and impressive rate performance. The results of this work can also be applied to the scalable manufacturing of other MXene-based flexible hybrid and nanocomposite films.

### 4.3. Sensors

With the development of wearable devices, artificial intelligence, and medical diagnosis, the demand for high-performance sensors is growing. The performance of the sensors largely depends on the flexibility, conductivity, and sensitivity of the materials. MXenes are widely used in flexible sensors due to their 2D layer structure, high conductivity, hydrophilicity, and huge specific surface area [108]. Currently, many factors, including surface-terminated groups (for instance, oxygen-containing), surface functions and dopants, and structural defects, have already been demonstrated to have a significant impact on the sensor performance [109]. The construction of MXene-based heterostructures can significantly improve the performance of sensors.

**Figure 10:**
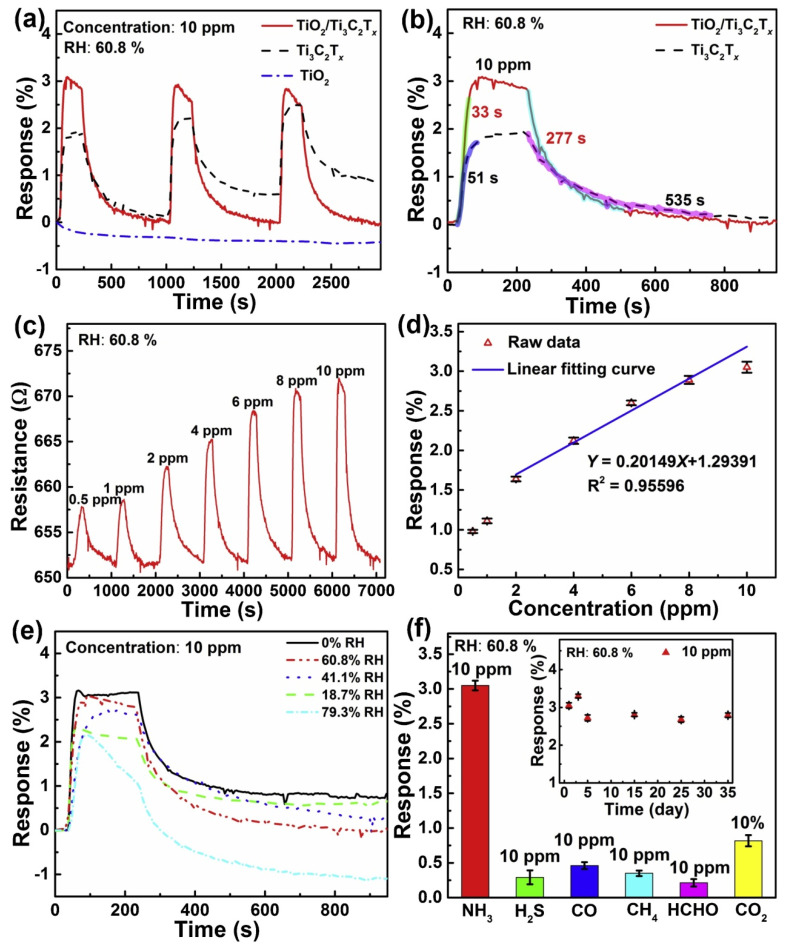
Performance of MXene−based sensors. (**a**) Comparison of the normalized response–recovery performance between the TiO_2_/Ti_3_C_2_T_x_, Ti_3_C_2_T_x_, and TiO_2_ gas sensors at a condition of 10 ppm NH_3_; (**b**) comparison of the response–recovery times between the Ti_3_C_2_T_x_ and TiO_2_/Ti_3_C_2_T_x_ sensors; (**c**) dynamic response−recovery curve of the TiO_2_/Ti_3_C_2_T_x_ gas sensor to different concentrations of NH_3_; (**d**) relationship between the response versus NH_3_ concentrations; (**e**) influence of RH on the response–recovery curves of the TiO_2_/Ti_3_C_2_T_x_ gas sensor under 10 ppm NH_3_; and (**f**) influence of gas types on the responses of TiO_2_/Ti_3_C_2_T_x_ gas sensor, inset gives the long-period stability of the sensor to 10 ppm NH_3_. Operating temperature: 25 °C. Reprinted with permission from Ref. [112]. 2019, Elsevier.

Recently, Zhang et al. [110] prepared Ti_3_C_2_@N-C heterostructures for an electrochemical sensor to detect heavy metals in seawater and tap water. The sensor showed excellent sensing performance with a low detection limit, high resolution, and excellent selectivity, which provides a good example for improving the electrochemical performance of Ti_3_C_2_-MXene. Gasso et al. [111] fabricated a gas sensor based on a MXene/SnO_2_ heterostructure synthesized by the hydrothermal method for detecting NO_2_ at room temperature. The detection of NO_2_ by this sensor-based SnO_2_/MXene heterostructure showed high selectivity, sensitivity, repeatability, reproducibility, and a stable sensing response. The construction of MXene-based heterostructures is an effective way to improve the sensing performance of gas sensors. Tai et al. [112] fabricated TiO_2_/Ti_3_C_2_T_x_ heterostructures used for the gas sensor, which was used to enhance the NH_3_ sensing performance of Ti_3_C_2_T_x_ nanosheets. The results showed that the TiO_2_/Ti_3_C_2_T_x_ sensor exhibited a larger response value (1.63 times) and shorter response/recovery times (0.65/0.52 times) than those of a pure Ti_3_C_2_T_x_ sensor to 10 ppm NH_3_ at room temperature of 25 °C (60.8% relative humidity) (Figure 10). Similarly, He et al. [113] also prepared a gas sensor based on a MXene/SnO_2_ heterostructure for NH_3_ detection. Different from Gasso’s research, this study focused on the wireless sensor made of an MXene/SnO_2_ heterostructure. The wireless sensor exhibited high sensitivity, rapid recovery, and a stable sensing response, which was attributed to the good conductivity and the charge transfer at the interface of MXene/SnO_2_ heterostructures. This research expanded the practical application of the gas sensor. Although the sensors made of MXene-based heterostructures have shown high selectivity, sensitivity, repeatability, and a stable sensing response, there are still some challenges needed to be overcome. Miniaturization, flexibility, passive wireless, and sensor fusion are the four development trends forsensor technology breakthroughs in the future.

### 4.4. Photocatalysts

In recent years, MXene has attracted much attention as a highly efficient photocatalyst [114]. However, MXene is usually not used as a photocatalyst by itself because of the rapid recombination of photocarriers and large surface area, which causes the particles to be easily aggregated onto MXene. Constructing a heterostructure is considered to be an effective method to improve the catalytic performance of MXene-based photocatalysts [115].

With the combination of MXene heterostructures with suitable photocatalysts, the photocatalytic performance of electron transfer can be greatly improved. Zhou et al. [116] prepared a ZnO/MXene heterostructure based on accordion-shaped MXene by electrostatic self-assembly and used it asa photocatalyst for dye degradation. Compared with pure ZnO, the photocatalyst-based ZnO/MXene heterostructure exhibited excellent photocatalytic performance and stable cyclic degradation, which may be because the heterostructures between ZnO and MXene shorten the carrier transfer path, facilitating the transfer of photogenerated electrons from the conduction band (CB) of ZnO to the CB of MXene. Chen et al. [117] designed and prepared unique 2D/2D CdS NS@Ti_3_C_2_ MXene heterostructures for photocatalytic hydrogen production. The experiment onphotocatalytic hydrogen production showed that the heterostructures showed a higher photocatalytic performance (1.73 mmol·h^−1^·g^−1^) than pure CdS NSs (0.37 mmol·h^−1^·g^−1^), which could be attributed to the high surface area and enhanced charge separation activity between CdS and Ti_3_C_2_ MXene.

**Figure 11:**
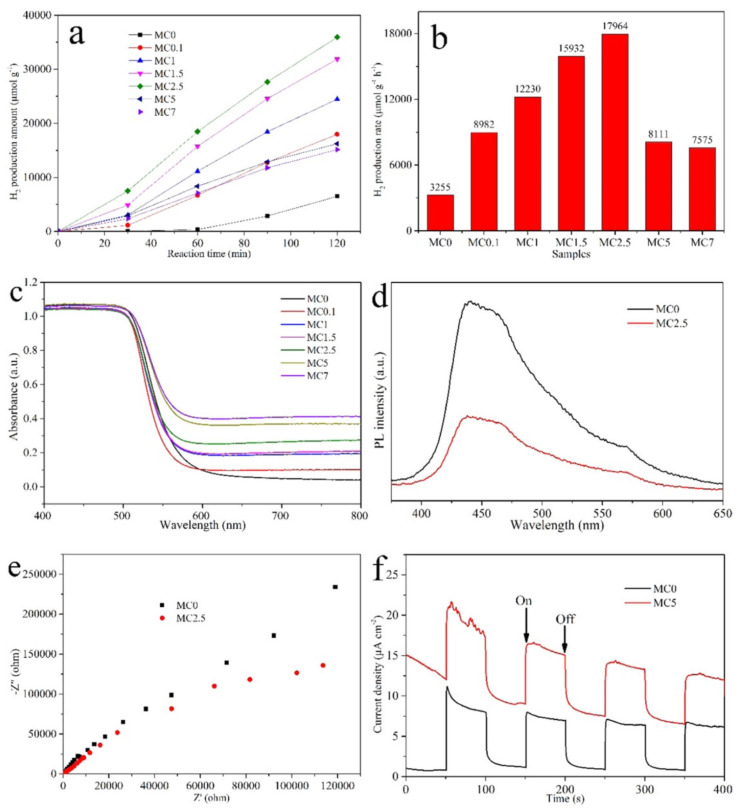
Photocatalytic performance of the MXene–based heterostructure. (**a**) Amount of H_2_ production regarding the reaction time for Mo_2_C/CdS heterostructures. (**b**) Average H_2_ production rate under different photocatalysts. (**c**) Performance of UV–vis diffuse reflectance spectra (DRS). (**d**) Performance of PL spectra for samples MC0 and MC2.5. (**e**) Performance of EIS plots for MC0 and MC2.5 electrodes under the condition of open–circle potential and visible light irradiation with 0.5 M Na_2_SO_4_, respectively. (**f**) Performance of TPC response of MC0 and MC2.5 electrodes under visible light irradiation with 0.5 M Na_2_SO_4_. Reprinted with permission from Ref. [119]. 2021, American Chemical Society.

In practical application, the photocatalytic efficiency of the binary photocatalysis system is still insufficient due to the challenges in inducing the carrier migration of separation and participating in redox reactions in some cases. Therefore, heterostructures are prepared by coupling other composites with cheap MXene conductive materials to induce charge transfer, and the charge carrier separation is improved by providing a larger reaction area, so as to improve the photocatalytic efficiency. Yang et al. [118] synthesized a novel ternary Ti_3_C_2_ MXene@TiO_2_/CIS (M@T/CIS) heterostructure photocatalyst by a two-step hydrothermal process for efficient photocatalytic H_2_ evolution. The M@T/CIS photocatalyst exhibited a high H_2_ evolution rate of 356.27 μmol·g^−1^·h^−1^, which was about 69.5 and 636.2 times higher than that of the Ti_3_C_2_ MXene@TiO_2_ (M@T) and CIS sample, respectively. Jin et al. [119] constructed Mo_2_C-MXene/CdS heterostructures as photocatalysts by the hydrothermal method. The experiment of photocatalytic hydrogen production showed that the photocatalyst showed excellent hydrogen production performance compared with the previously reported Ti_3_C_2_ MXene catalyst. Figure 11 shows the photocatalytic performance of the heterostructures. The author explained that Mo sites in the Mo_2_C-MXene/CdS(110) heterostructure provide an efficient reaction site for the hydrogen evolution reaction and this heterostructure has a favorable band gap and band edge position for water splitting. In addition, Khan et al. [120] constructed CoAlLa-LDH/g-C_3_N_4_ heterostructures based on single-layer Ti_3_C_2_ MXenes as photocatalysts to reduce CO_2_ to solar fuels. In the process of dry reformingof methane, the CO and H_2_ produced by the heterostructure under visible light were 55.25 and 54.72 μmol·g^−1^·h^−1^, respectively. The remarkable performance of the photocatalyst was due to the strong interface interaction formed by CoAlLa-LDH/g-C_3_N_4_ heterostructures to achieve excellent charge-transfer separation.

From the above research progress, it is seen that the MXene-based heterostructure has a considerably higher specific surface area when compared to a single MXene, which allows more active sites and provides more photoreactionsites, endowing the prepared photocatalysts with a stronger light capture ability and faster charge-transfer rate, so as to improve the photocatalytic reaction rate.

To summarize, this section mainly discusses four applications of MXene-based heterostructures. In the application of supercapacitors, the construction of heterostructures can expand the ion storage space and improve the electrochemical behavior. Pure MXene material is seriously stacked in the application of energy storage batteries. However, the heterostructure can provide a stable volume buffer space, and can make full use of the properties of the two components to improve the electrochemical performance. In addition, surface-terminated groups, dopants, and structural defects seriously affect the sensing performance of MXene-based sensors. The MXene-based heterostructures can improve the interfacial charge-transfer efficiency. The sensor based on MXene heterostructure has high sensitivity, rapid recovery, and a stable sensing response. In the application of photocatalysts, other composites are coupled with cheap MXene materials to prepare heterostructures and improve charge carrier separation by providing a larger reaction area, so as to improve photocatalytic efficiency.

## 5. Conclusions and Prospects

In this review, we discussed the recent research progress in the structure, fabrication, and application of MXene-based heterostructures. This paper introduced two kinds of MXene-based heterostructures: vertical structure and lateral structure. Then, three major synthesis methods for constructing MXene-based heterostructures were discussed, including the hydrothermal method, electrostatic self-assembly method, and chemical vapor deposition. The advantages and disadvantages of the three methods and how the preparation methods affect the performance of MXene-based heterostructures were also summarized. In addition, the applications of MXene-based heterostructures, such as supercapacitors, sensors, batteries, and photocatalysts, were discussed. Then, the role of MXene-based heterostructures in four applications was analyzed. MXene-based heterostructures can integrate the advantages of different materials to achieve complementary properties far beyond that of a single material. Nowadays, increasing research attention is attracted onto MXene-based heterostructures.

Although significant progress has been achieved, some challenging issues still exist. Firstly, the current synthesis method is time-consuming and labor-intensive, and the product quality is not good. It is necessary to further optimize the synthesis, namely, regulate the content, shape, and size of each component in the MXene-based heterostructure. Additionally, 3D printing technology such as inkjet printing can be used to manufacture large-scale heterostructures, and the preparation process is less time-consuming and efficient, which can be considered as one of the promising methods to fabricate MXene-based heterostructures in the future. Meanwhile, various theoretical predictions of MXene-based heterostructures need to be further confirmed in future experiments to broaden the application potential. In addition, although MXene-based heterostructures have been widely used, the issues such as agglomeration, re-stacking, and oxidation of MXene nanosheets commonly exist, which significantly hinder their performance. An in-depth understanding of the various factors that influence them, such as surface chemistry, interlayer structure, and layer stacking, is helpful to fabricate high-performance heterostructures.

In conclusion, the study of MXene-based heterostructures is promising. After the challenges are resolved, more types of MXene-based heterostructures can be designed in the near future. The applications of MXene-based heterostructures may be greatly promoted.

## Data Availability

The data that support the findings of this study are available from the corresponding authors upon reasonable request.
